# The Effects of Lead Species and Growth Time on Accumulation of Lead in Chinese Cabbage

**DOI:** 10.1002/gch2.201600020

**Published:** 2017-02-15

**Authors:** Megan Corley, Samuel Mutiti

**Affiliations:** ^1^ Department of Biological and Environmental Sciences Georgia College and State University CBX 081 Milledgeville GA 31061 USA

**Keywords:** accumulation, bioaccumulation, bioremediation, heavy metals, plants

## Abstract

A major pathway for heavy metal exposure in contaminated areas is via consumption of locally produced food. This study investigated the accumulation of lead in Chinese cabbage grown in contaminated soils and estimated the weekly dietary intake. Experiments were conducted to determine the effects of different growth times, concentrations, and lead species (carbonate, nitrate, and sulfide) on the uptake of lead in shoots. Results show that Chinese cabbage accumulated up to 38 mg kg^−1^ in the shoots. There was a significant difference in lead uptake by plants grown in soils with 400 mg kg^−1^ (Upper Critical Limit: UCL) and those grown in 600 mg kg^−1^ (Above Critical Limit: ACL) lead concentrations. However, there was no significant difference in the ACL shoots despite the different growth period. The cabbages grown for eight weeks (at UCL) had four times more lead than those grown for four weeks. The elemental form also affected lead uptake with the lead sulfide (mineral form) having the least uptake and lead carbonate (solution) having the highest. Calculated weekly dietary intake levels of lead were higher (above 0.28 mg kg^−1^ per human body weight) than the recommended levels for human consumption (0.025 mg kg^−1^ per human body weight).

## 1 Introduction

Human exposure to high concentrations of heavy metals, such as lead, can cause severe health problems and, in some cases, even death. People living in heavily polluted areas, especially near current and former lead and zinc mines, smelters, or superfund sites, are at high risk of exposure to these heavy metals in their daily lives. One of the most adversely affected mining towns in the world is the town of Kabwe located just south of a well‐known mining region in Zambia.^1^, ^2^, ^3^, ^4^, ^5^, ^6^ Kabwe was host to a very productive lead, cadmium, and zinc mine for nearly 90 years before the closure of the mine in 1994.^4^ Lead sulfide was the main lead ore in a carbonate host rock.^7^ As a result of lax environmental regulations that did not limit emissions into the environment from the mine during the period of mine operations, widespread heavy metal contamination has caused this town to be listed among the top ten worst polluted places in the world [http://www.worstpolluted.org]. Lead concentrations in the local soils are extremely high, ranging from 700 to 42 000 mg kg^−1^,^2^, ^4^, ^6^, ^8^, ^9^ which is above the US Environmental Protection Agency's (EPA) recommended safe levels (upper critical limit, UCL) of 400 mg kg^−1^ [http://www.epa.gov/region1/leadsafe/pdf/entire_document.pdf].

Lead exposure can cause a number of diseases including hematological, gastrointestinal, and nephropathy problems [http://www.epa.gov/superfund/lead/health.htm]. Previous studies conducted in Kabwe have shown that the blood concentrations in children that play in the contaminated soils are elevated and can reach up to 200 µg dL^−1^ with the average being from 60 to 120 µg dL^−1^.^2^ The current Center for Disease Control (CDC) recommended levels of lead in children's blood is 5 µg dL^−1^ and the EPA has shown that after 10 µg dL^−1^ lead begins to cause diseases and disabilities in children [http://www.cdc.gov/nceh/lead/default.htm; http://www.epa.gov/region1/leadsafe/pdf/entire_document.pdf]. Exposure to these heavy metals in the area is not only from direct contact with soil, but also from ingesting meats and vegetables produced or grown in the region.

Studies from different parts of the world have revealed that vegetables grown in contaminated soils can accumulate unhealthy amounts of lead in the edible parts of the plant. Sangster et al.^10^ found that leafy greens grown in a community garden of Nebraska, USA were able to accumulate significant amounts of lead (16 mg kg^−1^), well over the tolerable intake level for lead set by the FDA [<1 mg kg^−1^; http://www.fda.gov/Food/FoodborneIllnessContaminants/Metals/ucm233520.htm]. A study in France^11^ also showed that vegetables grown in contaminated soils near a former smelter had significant lead levels to be considered a health hazard, with children being the most at‐risk group. A study by Guo et al.^12^ in China showed that industrial activities were resulting in heavy metal contamination of nearby agricultural fields. Cabbage, lettuce, and other vegetables grown in these fields accumulated significant concentrations of lead metal with cabbage and lettuce accumulating about 22 mg kg^−1^. In Kabwe, Nwankwo and Elinder^3^ investigated the presence of lead in corn, spinach, cabbage, and rape grown. Their results showed high levels of lead in these vegetables (20.5–322 mg kg^−1^). At these levels of lead contamination, the vegetables are considered heavily contaminated and could potentially be detrimental to human health. Based on these findings, it is possible that other vegetables grown in the area could also be dangerously contaminated. Vegetables commonly grown in the backyard gardens include carrots, onions, rape, and Chinese cabbage.

For heavily contaminated areas, vegetables are not the only food products that can be tainted with lead and other heavy metals (such as copper, cadmium, and zinc). Yabe et al.^13^ found that cattle raised in the Kabwe area had elevated concentrations of lead and other heavy metals in the liver and kidney meat that is consumed by the local people. They recommended reducing consumption of these meats (specifically by children and pregnant women) and monitoring lead levels in cattle. A few studies have recommended investigating limiting lead exposure through changes in dietary habits, therefore reducing how much lead is being directly ingested into the body. Ingesting any form of lead, even in small amounts, is dangerous and can accumulate in the body, usually in the tissue or bones [2; http://www.epa.gov/superfund/lead/health.htm]. This is especially true for children that often are at higher risk for lead poisoning.

The purpose of this study was to investigate the uptake of lead by Chinese cabbage (*Brassica chinensis*) when grown in soils contaminated with different concentrations and three lead species. The primary goal was to assess the accumulation of lead in Chinese cabbage when grown in soils contaminated with concentrations around the lead upper critical limit concentration (400 mg kg^−1^) and grown for different periods of time. A secondary goal was to compare the uptake of three lead species; lead nitrate, carbonate, and sulfide. We hypothesized that Chinese cabbage will take up more lead in the shoots when grown in soils contaminated with lead nitrate than when grown in lead carbonate or lead sulfide contaminated soils. The results were then used to evaluate how much lead contamination a person would be ingesting weekly if they ate Chinese cabbage grown in contaminated soils. The project results will be used to inform the residents on what quantities of Chinese cabbage they can safely consume if grown in local soils that are heavily contaminated with lead.

## 2 Results

The lead concentrations in the control soils ranged from 25 to 31 mg kg^−1^ and averaged 28 mg kg^−1^. Soils for the 400 mg kg^−1^ treatment ranged in lead concentrations from 338 to 490 mg kg^−1^ and averaged 410 mg kg^−1^. For the 550 mg kg^−1^ treatment soils had lead concentrations ranging from 482 to 579 mg kg^−1^ with an average of 540 mg kg^−1^. Lead concentrations for the 600 mg kg^−1^ treatment ranged from 507 to 686 mg kg^−1^ and averaged 603 mg kg^−1^. There was no statistical difference between the 550 and 600 mg kg^−1^ treatments (*F*_1, 10_ = 3.75, *P* = 0.08 > 0.05).

**Figure 1:**
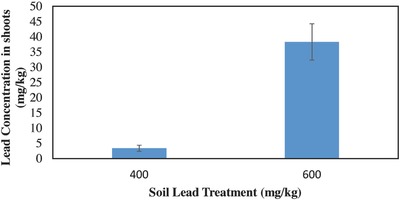
After four weeks of growth, the lead concentrations in the shoots were significantly higher in the 600 mg kg^−1^ lead soils than the UCL lead soils.

**Figure 2:**
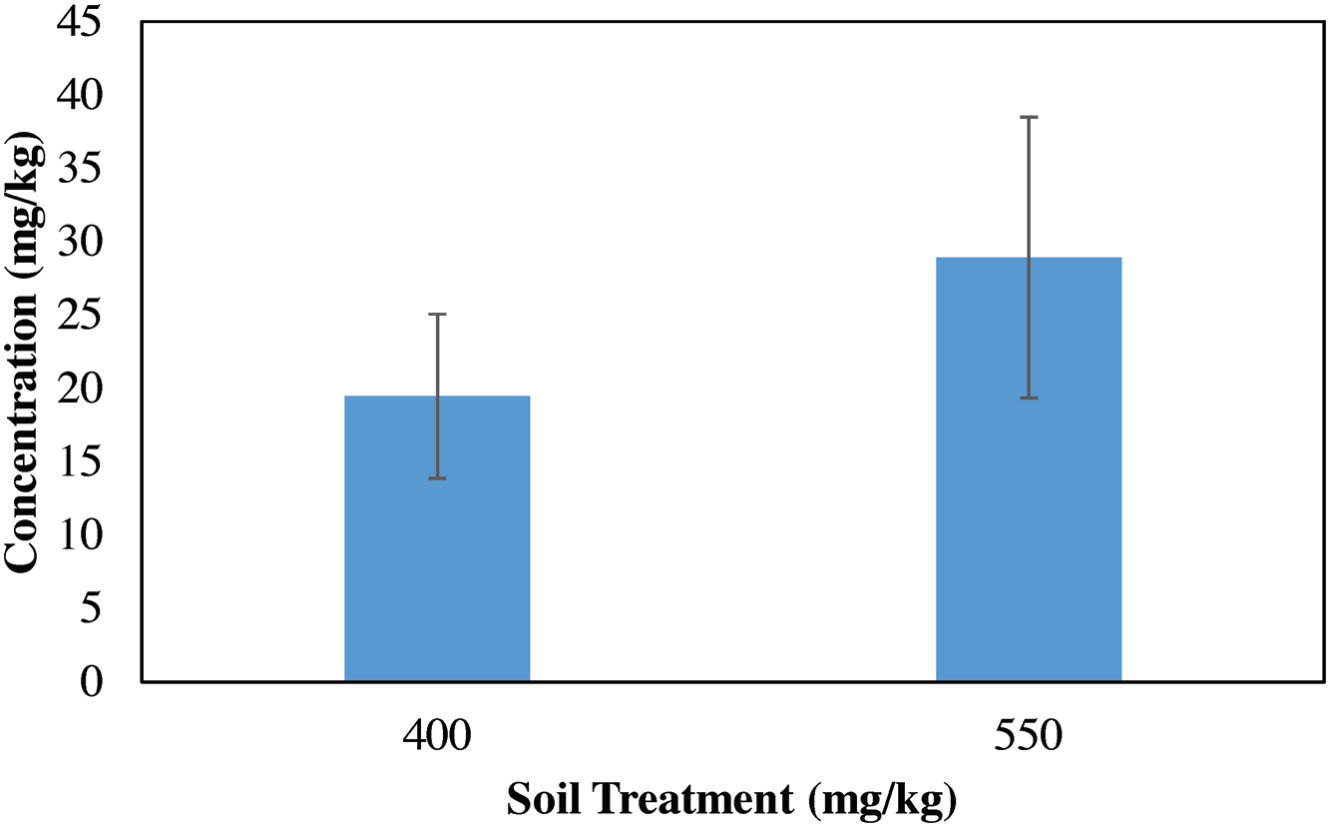
No statistically significant difference in shoot lead concentrations after eight weeks of growth in soils contaminated with 400 and 550 mg kg^−1^ lead treatment.

Chinese cabbage grown for four and eight weeks in lead concentrations ranging from 400 to 600 mg kg^−1^ were only able to accumulate an average of 20 mg kg^−1^. The highest concentration measured was 38 mg kg^−1^ in the shoots of plants grown for four weeks in 600 mg kg^−1^ lead soil treatment. Lead concentrations in Chinese cabbage differed significantly between the UCL and the 600 mg kg^−1^ soil treatments after four weeks (*F*_1, 8_ = 27.9, *P* = <0.001), **Figure** **1**. Chinese cabbage grown in the higher concentration took up significantly more lead in the shoots than the plants grown in the lower concentration. After eight weeks of growth, lead concentrations did not differ significantly between UCL soil treatment and the 550 mg kg^−1^ soil treatment (*F*_1, 10_ = 0.73, *P* = 0.41 > 0.05), **Figure** **2**.

**Figure 3:**
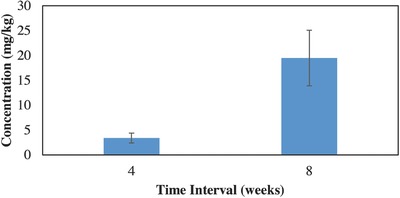
A significant difference was observed between lead concentrations after four weeks of growth and eight weeks of growth of the UCL lead soil contamination.

**Figure 4:**
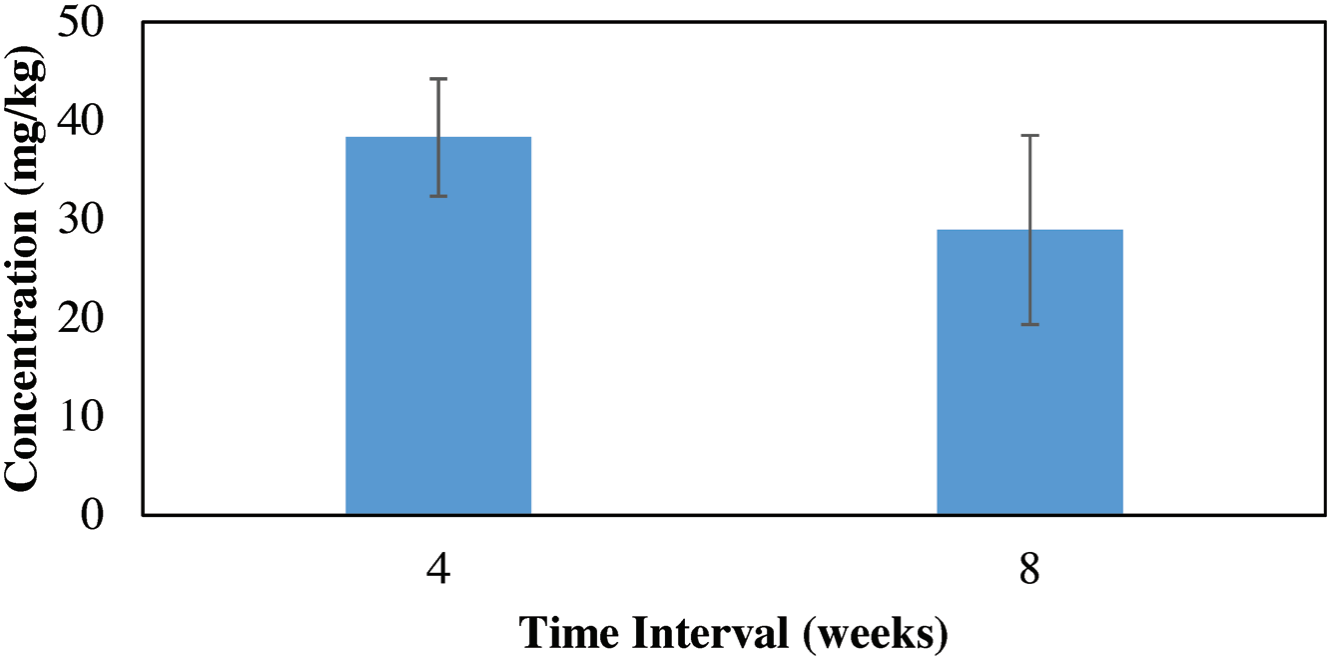
Comparing shoot lead concentrations after four and eight weeks of growth of the above UCL lead soils showed no statistically significance difference between the two growing times.

For the plants grown in the lower concentrations (UCL), shoot concentrations were significantly different between four and eight weeks (*F*_1, 9_ = 6.62, *P* = 0.03 < 0.05). The four additional weeks of growth time resulted in the UCL treatment plants taking up four times as much lead as those grown for four weeks at the same treatment concentration (**Figure** **3**). For plants grown in higher lead concentrations (>550 mg kg^−1^) shoot concentrations did not differ significantly between the four‐ and eight‐week growing periods (*F*_1, 9_ = 0.60, *P* = 0.46 > 0.05), **Figure** **4**. With more time, the plants grown in the higher concentrations were not able to take up any more lead than the concentrations at four weeks. Not only were the plants not able to take up more, but the mean shoot concentrations decreased slightly between the four‐ and eight‐week harvest period (Figure 4).

**Figure 5:**
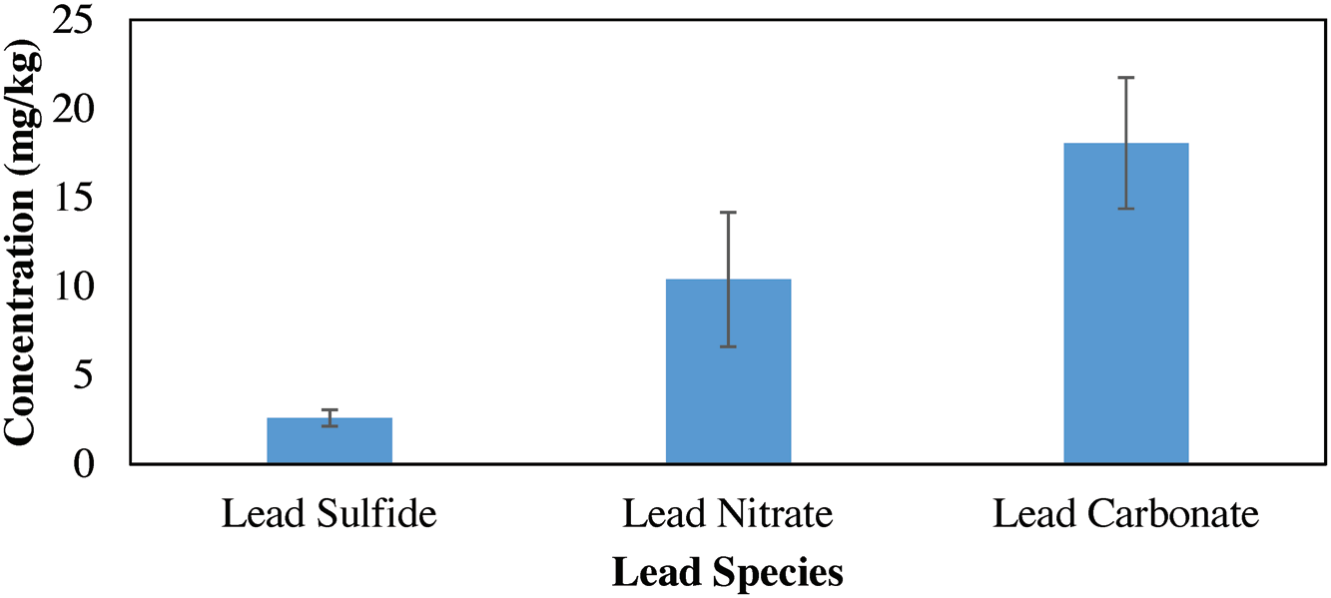
Comparison of lead nitrate, lead carbonate, and lead sulfide uptake in Chinese cabbage showed more lead in the carbonate and lowest in the sulfide.

Plants in the carbonate treatment appeared to take up more lead (had a higher mean) than the nitrate treatment but there was no statistical difference between the two treatments (*F*_1, 24_ = 2.13, *P* = 0.16 > 0.05). There was also no statistically significant difference between lead nitrate and lead sulfide treatments (*F*_1, 21_ = 3.38, *P* = 0.08 > 0.05). Plants grown in the lead nitrate treatment had a higher mean than the plants in the lead sulfide treatment, but lower than the lead carbonate treatment (**Figure** **5**). There was a significant difference in shoot concentrations between the lead carbonate and the lead sulfide treatments (*F*_1, 21_ = 12.7, *P* = 0.002 < 0.05). The average concentration of lead in the shoots of the Chinese cabbage grown in the lead carbonate treatment was about 18 mg kg^−1^, in the lead nitrate treatments the concentration was about 10 mg kg^−1^ and in the lead sulfide treatments the concentration was about 3 mg kg^−1^. One additional finding when comparing plants grown in different lead species was the definite difference in plant biomass production. The plants grown in lead nitrate produced more biomass (average dry biomass of 3.0 g) and looked healthier than those grown in either the lead carbonate (2.0 g) or lead sulfide (2.0 g) treatments.

The enrichment coefficient (EC) values from all lead contaminated treatments were less than one as shown in **Table** **1**. Chinese cabbage grown in the 600 lead nitrate treatment had the most uptake of lead from the soils, but the EC values were still relatively low.

**Table 1:** Enrichment coefficient of Chinese cabbage ranging from 0.001 to 0.06 for all the plants. Here LN is lead nitrate, LC is lead carbonate, and LS is lead sulfide

Four weeks		Eight weeks		Ten weeks	
Treatment [ppm]	EC	Treatment [ppm]	EC	Treatment [ppm]	EC
400 LN	0.007	400 LN	0.049	1500 LC	0.013
600 LN	0.064	550 LN	0.053	1500 LN	0.009
				2000 LS	0.001

## 3 Discussion

Concentrations of lead in Chinese cabbage shoots (Figures 1 and 2) are equivalent or higher to the concentrations that Chinese cabbage and other similar leafy vegetables have been shown to take up from lead contaminated soils in other studies.^3^, ^10^, ^11^, ^15^, ^17^ Chinese cabbage grown in soils with higher concentrations (>400 mg kg^−1^) reached the maximum accumulation concentration at four weeks and decreased to a slightly lower concentration at eight weeks (Figure 4). This decrease of heavy metals in the plants over time was also shown in another.^16^ Adesodun et al.^16^ showed that after four weeks lead concentrations in sunflowers decreased and continued to decrease with time, indicating that the optimal time to harvest sunflowers for phytoremediation would be after the initial growth period of four weeks. The decrease in concentration over time shown in this study could be attributed to a decrease in lead accumulation. The plants appeared to reach a maximum accumulation concentration while biomass production continued, resulting in the same total lead accumulated but a lower concentration (lead mass/plant mass) in plants with more biomass. Results from this study, even though preliminary, show that when Chinese cabbage is grown in soils contaminated with lead at UCL concentrations, they should be harvested as soon as possible in order to reduce lead heavy metal exposure. However, for Chinese cabbage grown in soils contaminated with lead equal to or greater than 550 mg kg^−1^, waiting longer to harvest could potentially reduce exposure to lead. It appears that the most heavy metal exposure to humans (via consuming Chinese cabbage grown in highly contaminated soils) could be from harvesting the shoots immediately when the plants are large enough to harvest (at about four weeks). The cabbage seem to rapidly reach a maximum accumulation concentration, while biomass production continues resulting in a low total lead to biomass ratio, hence the need to wait past the four weeks' maturity stage. More research is still needed to see how consistent these observations would be with this and other varieties of Chinese cabbage.

Results from this study also indicate that the solubility of the lead species (when contamination is in soluble form) does not affect the bioavailability of lead to Chinese cabbage. This does not support our original hypothesis that the low solubility of the carbonate species would make it less available to the plants and therefore would not be taken up in amounts detrimental to human health. The plants seem to be able to access the lead just as easily from the lead carbonate compared to the lead nitrate. Chinese cabbage in this study also appeared to grow consistently better in lead nitrate treatments than either the lead carbonate treatment or the lead sulfide treatment despite all plants receiving sufficient and similar additional nutrients (fertilizer). This could be a result of more nitrates being released by the lead nitrate in the soil, therefore being more available to the plants. However, these observations need further investigation to verify this hypothesis. The plants took up more and accumulated higher concentrations of lead carbonate than lead sulfide (Figure 5). Lead sulfide had the least accumulation of lead in the shoots most likely due to it being added to the soils as the mineral galena. Even though there was an incubation period, the lead sulfide was not as readily available to the plants as the other lead treatments that were in a powder forms.

This study did not use soils from a contaminated field site because Hamvumba et al. had already conducted such a study using soils from the Kabwe site. Their results showed much higher lead uptake in Chinese cabbage than this study. In soils contaminated with only 58 mg kg^−1^ total lead (10 mg kg^−1^ extractable lead) Chinese cabbage took up over 20 mg kg^−1^ in the shoots.^15^ The main lead minerals in Kabwe soils are expected to be cerussite (carbonate) and galena (sulfide). These are the two main ore minerals of lead in Kabwe, which also has minor compositions of pyromorphite, mottramite, vanadinite, and descloizite.^7^ Comparing results from this study to those from Hamvumba et al.,^15^ it can be inferred that the lead species produced after incubation in this study were less bioavailable than the species found in the soils in Kabwe. The soils had similar cation exchange capacity (CEC) and pH values. Further studies need to be carried out to determine the actual lead mineralization in the lab soils after incubation and those in the Kabwe soils.

Despite the consistent uptake of lead in Chinese cabbage, EC values were all below the hyperaccumulator status threshold value of 1. When EC values are greater than 1, a plant is considered efficient at taking up that specific heavy metal and a possible hyperaccumulator species.^16^ Results from this present study show that Chinese cabbage is not a hyperaccumulator of lead and, therefore, not a good candidate for phytoremediation. Although the shoots did not take up enough lead to make Chinese cabbage a good candidate for phytoremediation, the amounts of lead taken up in the shoots were not safe for human consumption. This means that people growing Chinese cabbage in heavily contaminated areas are at high risk of ingestion of dangerous lead levels. Many vegetables, including Chinese cabbage, are commonly sold at local markets and grown in residents' backyards in Kabwe, Zambia. These vegetables are consumed multiple times a week and are a staple in the diets of most people living in the area. From the levels of contamination found in this study, a weekly dietary intake of lead was assessed for how much lead metal an average person would be exposed to if their diet included Chinese cabbage grown in soils contaminated with the different lead species. **Table** **2** shows the dietary intake measurements that were calculated based on the formulae from the study of Krˇíbek et al.^18^ of the dietary intake of lead from cassava roots and leaves.

**Table 2:** Calculated dietary intake of lead assuming consumption of 1 kg of Chinese cabbage weekly and a human body weight (HBW) of 70 kg were all above the 0.025 mg kg^−1^ per HBW. Here LN is lead nitrate, LC is lead carbonate, and LS is lead sulfide

Treatment	Concentration[mg kg^−1^]	Weekly lead intake[mg kg^−1^ per HBW]
LN 400	19.48	0.28
LC 1500	18.08	0.26
LS 2000	2.62	0.04

The provisional tolerable weekly intake for lead is 0.025 mg kg^−1^ per human body weight (http://jecfa.ilsi.org). Based on our estimated weekly intakes in Table 2, Chinese cabbage grown for four and ten weeks in lead contaminated soils was well over the provisional tolerable weekly intake for lead. If someone were to consume a significant amount of Chinese cabbage grown in soils contaminated with 400 mg kg^−1^ or greater they would be exposed to significant health risks over their lifetime. If the soil concentrations have more lead contamination than the values used in this study, which is the case for garden soils in Kabwe, then people living in the area are consuming unhealthy quantities of lead on a weekly basis. For a place like Kabwe, and most polluted areas in the world, other vegetables grown in local soils and livestock in the area will also take up significant amount of lead.^3^, ^13^, ^19^ This is cause for great concern because most of their locally produced foods are potential pathways for heavy metal exposure to humans.

### 3.1 Best Management Practices Recommendations

After analyzing data from this study and other studies about lead pollution in communities all of the world, the authors would like to make the following recommendations for people living in lead‐contaminated areas:

- Have garden soils and water tested for lead and other heavy metals
- Have all commonly grown vegetables in the area assessed for their potential to accumulate lead and other heavy metals
- Create daily or weekly maximum consumption guides for different vegetables or combination of foods grown in contaminated soils
- Limit consumption of food grown or produced in heavily contaminated soils
- Whenever possible use raised beds with clean soils or a mixture of local soils with clean soils to grow vegetables
- Use dust shields on the windward side of gardens to protect vegetables from dust emanating from the most contaminated parts of the area
- Thoroughly wash hands before handling food and wash vegetable with clean water
- Monitor blood levels in people, especially children, and whenever possible implement natural strategies for reducing lead levels when higher lead levels are detected in blood samples (http://documents.worldbank.org/curated/en/2011/10/15583313/zambia‐copperbelt‐environment‐project).

People consuming leafy vegetables grown in contaminated soils are at higher risk of serious lead exposure. Chinese cabbage grown in lead‐contaminated soils will accumulate significant amounts of lead regardless of the lead species found in the soil. This is especially important for children whose bodies are more capable of long‐term storage of lead within the tissue and bones. Reducing consumption of locally grown Chinese cabbage and leafy vegetables could help reduce the overall lead exposure to the people in living in contaminated areas. Chinese cabbage can be effectively grown in contaminated soils from both seeds and seedlings without significant problems. In the future, other vegetables will also be grown in contaminated soils to determine the potential of heavy metal accumulation in the shoots. The main vegetables that will be used are vegetables commonly grown and eaten in the Kabwe area and will include carrots and onions so that a safe weekly intake amount can be determined.

## 4 Conclusion

This paper addresses the issue of lead exposure through consumption of contaminated vegetables grown in polluted areas. Heavy metal exposure is a major concern in the world today and one that needs our attention. This work falls under the agriculture, food, and global health challenges. To the authors knowledge this is the first time the effect of lead mineral form (species) on the ability of the vegetables to take up lead has been assessed. It is also the first time the weekly dietary intake levels for Chinese cabbage grown in contaminated soils have been evaluated, and best management practices for people growing plants in polluted sites presented. Results from this study could potentially help reduce human exposure to lead via consuming contaminated Chinese cabbage and thereby increase general health, productivity, and longevity. This study also shows that thorough washing of vegetables is a just an initial step to reducing exposure but is not the solution as the metal is taken up into the plant tissue. Removing the soils or growing the vegetables in unpolluted soils is key to reducing exposure. Knowledge of how the mineralogy affects uptake in the cabbage will guide remediation of polluted sites, especially around people's homes and gardens.

## 5 Experimental Section

*Experimental Procedure*: This study was conducted in a temperature‐controlled greenhouse using locally acquired topsoil. The soil was amended with sand to increase the mineral content as it was mostly composed of organic matter. The resulting CEC value was about 5.8 meq per 100 g, and pH values ranged from 5.2 to 6.8. Soils used in the contaminated treatments were incubated in water containing the various lead species for over one week to permit fixing of lead by the soils from the soluble forms to less soluble species, which occurs within a few days.^14^

Lead solutions were created by mixing either solid lead nitrate or lead carbonate in water (complete dissolution was not achieved so some of the lead was added to the soil still in solid form). Lead sulfide was added directly to the soil in the form finely powdered galena. Lead nitrate was used in this experiment because it is highly soluble compared to other lead species. Lead carbonate and sulfide were used as comparison species to the highly soluble lead nitrate because they are less soluble lead species, therefore should theoretically not be as readily bioavailable.^2^ Lead carbonate and sulfide are also commonly found mineral forms of lead in Kabwe,^7^ which makes them good models to utilize in this study. For this study lead sulfide was derived from the mineral galena while lead nitrate and carbonate were obtained in powder form (Sigma‐Aldrich). The choice to conduct pot experiments using nonmineral forms of lead nitrate and sulfide was made because Hamvumba et al.^15^ already conducted a study using soils that were potentially contaminated by their mineral forms.

After lead solutions had been made, soils were soaked in them to reach the desired treatment concentration upon air drying. Soils were allowed to incubate for over a week before seeds were planted. To study the effects of different soil concentration on plant uptake, four lead nitrate treatments were prepared: 0 mg kg^−1^ (a control), 400 mg kg^−1^ (lead upper critical limit), 550 mg kg^−1^, and 600 mg kg^−1^ lead contaminated. Twelve replicates at the UCL concentration were prepared so that six pots could be harvested after 28 d and the other six after 56 d. Twelve additional replicates were prepared above the UCL concentration to allow for six pots to be harvested after 28 d (600 mg kg^−1^) and six to be harvested after 56 d (550 mg kg^−1^). The 550 mg kg^−1^ was treated as equivalent to the 600 mg kg^−1^ treatment because it was intended to be 600 mg kg^−1^ and there was no statistical difference in lead concentrations of the soils between the two treatments. The final soils in this treatment averaged 550 mg kg^−1^ because some of the solid lead nitrate remained in the container during soil contamination. For this reason, all the comparisons done in this study were between 400 mg kg^−1^ and concentrations between 550 and 600 mg kg^−1^. For this part of the experiment, Chinese cabbage (*B. chinensis*) seeds, with a recommended growth time of 40 d, were planted directly into contaminated pots. This was done in order to observe how lead contaminated soils would affect germination of young plants in gardens. To assess the effect of different lead species on plant uptake, four different concentration treatments were also setup using all three lead species; 0 mg kg^−1^ as the control, 1500 mg kg^−1^ for lead nitrate and carbonate, and 2000 mg kg^−1^ for lead sulfide. Chinese cabbage plants (Napa type, with a 60 d maturity growing period) were bought already in their initial growth stages from a local store. Twenty six plants were transplanted into both the lead nitrate and lead carbonate contaminated soil treatments (13 in each metal species) and ten plants were transplanted into the lead sulfide contaminated soil treatments. In this portion the study, plants and soils were harvested and processed after 70 d of growth to allow for maximum growth.

During harvest, entire Chinese cabbage plants were uprooted and the shoots separated from the roots. Soil samples were also taken from individual pots in order to determine enrichment coefficients for each plant system. The soils were homogenized before sample collection to ensure that the sample was representative of the entire soil system. Plant shoot samples were washed thoroughly and then dried at 70 °C for 24–48 h together with the soil samples. After drying, the shoots were weighed to determine the dry weight.

*Analytical Procedure*: An Olympus Delta X‐ray fluorescence spectrometer (XRF) from Olympus (Boston, MA, USA) was used to quantify the total concentration of lead in the plant shoots and soils. Plant samples were crushed to a powder before being analyzed with the XRF. For verification and confirmation of results, lead concentrations in six of the plant and soil samples were also quantified using both graphite furnace and a flame atomic absorption spectrometer (AAS) depending on the concentration in the sample. Plant and soil samples for the AAS were digested using the Aqua Regia method to ensure total lead extraction from the samples (http://www.cdc.gov/niosh/docs/2003‐154/pdfs/7301.pdf). XRF results were within 10% of AA results and therefore the XRF was used for the rest of the samples in this study.

A two‐way ANOVA in SPSS Statistics Version 22 program (IBM, Armonk, New York, USA) was used to compare lead concentrations in the plants among different lead treatments and across the time intervals of four and eight weeks. The data analysis function in Microsoft Excel (Microsoft, Redmond, WA, USA) was additionally used to perform single factor ANOVA. Outliers in each data set were removed before analysis was performed. Enrichment coefficients were calculated for each replicate by dividing the shoot lead concentration by the soil lead concentration.^16^
